# Long term effect of primary health care training on HIV testing: A quasi-experimental evaluation of the Sexual Health in Practice (SHIP) intervention

**DOI:** 10.1371/journal.pone.0199891

**Published:** 2018-08-01

**Authors:** Kamla Pillay, Melissa Gardner, Allon Gould, Susan Otiti, Judith Mullineux, Till Bärnighausen, Philippa Margaret Matthews

**Affiliations:** 1 Homerton Hospital, London, United Kingdom; 2 Sexual Health in Practice Community Interest Company, London, United Kingdom; 3 Killick Street Health Centre, London, United Kingdom; 4 Whipps Cross Hospital, London, United Kingdom; 5 Public Health, London Borough of Haringey, London, United Kingdom; 6 Sexual Health Promotion, Birmingham, United Kingdom; 7 Africa Health Research Institute, Somkhele, South Africa; 8 Institute of Public Health, Heidelberg University, Heidelberg, Germany; 9 Infection and Population Health, University College London, London, United Kingdom; 10 Department of Global Health and Population, Harvard T.H. Chan School of Public Health, Boston, United States of America; 11 Division of Infection and Immunity, University College London, London, United Kingdom; 12 Africa Health Research Institute, Somkhele, South Africa; Harvard Medical School, UNITED STATES

## Abstract

**Background:**

To examine the effect of Sexual Health in Practice (SHIP) training for general practitioners (GPs) on HIV testing rates in Haringey, a deprived area of London, UK, with a population of over 250,000 and HIV prevalence of 0.7% (in 2014). SHIP is an educational intervention delivering peer-developed and peer-led face-to-face training to improve quality of sexual and reproductive health (SRH) care.

**Methods:**

We carried out a quasi-experimental study of intervention effects across 52 GP practices (2008–2016). We used time variation in SHIP intervention exposure for effect estimation, controlling for practice and calendar month fixed effects in panel analysis. From 2008–2010, baseline data were collected, and in the subsequent six-year period, 78 GPs in Haringey (approximately 40% of all GPs) were SHIP trained. 46 Haringey practices (of 52) had at least one trained doctor. Outcome measures were monthly HIV tests and results by practice (obtained from the hospital laboratories).

**Results:**

SHIP significantly increased HIV testing; for every GP trained, practice HIV testing rates increased by 16% (testing rate ratio (TRR) 1.16, 95% confidence interval (CI) 1.05–1.28, p value 0.004). This significant effect was demonstrated using an 8-year observation period, and was sustained over the post-intervention period. An average of 1.42% of HIV tests were positive.

**Conclusion:**

SHIP training produces a significant and sustained increase in HIV testing for each GP trained. Compared with general population screening, HIV tests used in routine clinical care have a high probability of detecting a positive person. Unlike an RCT, this evaluation is a ‘real life’ measure of the effect that commissioners of SHIP could expect in comparable areas of the UK. The effectiveness of the SHIP training may be related to the programme components not included in interventions that did not demonstrate an effect, such as peer-led teaching, and use of approaches to communication and rapid risk assessment tailored to the setting.

## Introduction

Despite highly effective treatment, HIV remains a major public health issue. Of the estimated 101,200 people infected in the UK, 13% are thought to remain undiagnosed.[1] Late HIV diagnosis is associated with significant mortality and increased risk of transmission.[2–4] ‘Treatment as prevention’ guidelines are considered unlikely to decrease HIV transmission in the UK unless individuals living with HIV are aware of their serostatus.[5] There is evidence to suggest that people with undiagnosed HIV visit their GPs, but that this opportunity for diagnosis may be missed.[6, 7] Primary health care therefore presents opportunities to increase diagnosis.

Current clinical guidance in the UK [8–10] gives numerous strategies to increase HIV testing in primary health care (S1 File). However the ‘implementation gap’, is well recognised and guidelines alone will not bring changes to clinical practice[11], including in general practice.[12] With respect to sexual health, the gap is harder to bridge[13–15] because of stigma.[16–19] Clinician, patient and system factors have each been found to impede STI and HIV testing.[16–19] However, educational interventions to increase GP chlamydia and HIV testing tend to be ineffective.[20–22] An ongoing theme is that interventions fail to overcome barriers to testing specific to this setting.[20]

**Fig 1:**
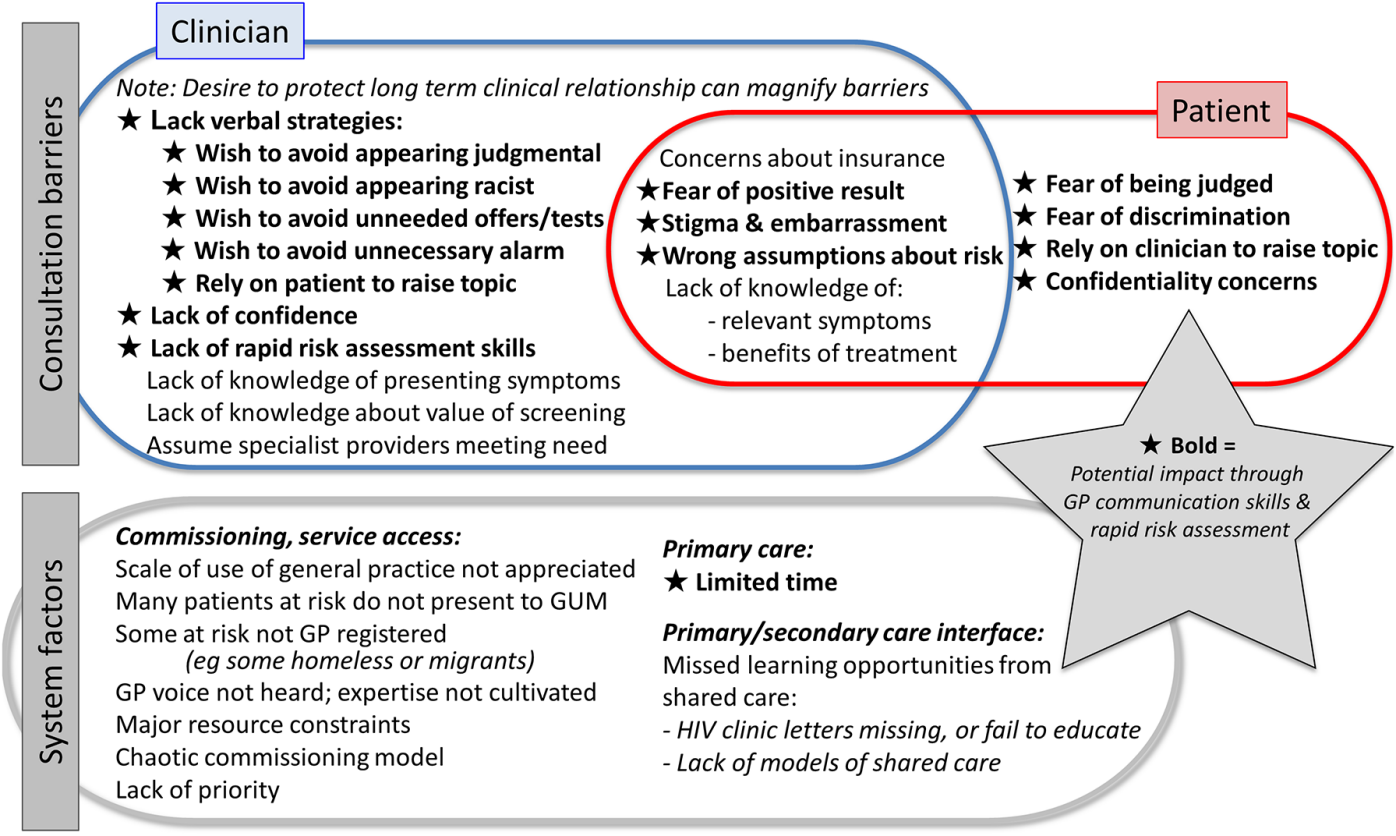
Barriers to HIV testing in primary health care.

Sexual Health in Practice (SHIP) is a peer-developed and -led educational intervention closely tailored to general practice that aims for broad improvement in sexual health care. In previous mixed methods evaluation of SHIP[23, 24] a range of effects were found. SHIP appears to differ from other interventions by tackling the barriers unique to the setting (Fig 1) by teaching specific verbal strategies and an approach to rapid risk assessment developed for general practice.

This quasi-experimental observational study measures the effectiveness of SHIP training of GPs in increasing HIV testing rates. We use practice fixed effects to control for both observed and unobserved time-invariant confounders of the relationship between the number of SHIP trained GPs and HIV testing. The effect of training differentiated by local HIV prevalence within Haringey and response to training beyond 6 months can both be evaluated. The effect of SHIP training of GPs and practice nurses is compared. We report positivity rates of HIV tests in routine clinical use in general practice. Lastly, we identify components of SHIP that differ from other interventions that may possibly explain the mechanisms of effect.

## Methods

### Study design and data collection

We used de-identified data on i) monthly practice-level exposure (SHIP trained GPs and nurses) and ii) the outcome (the numbers of HIV tests carried out by each practice each month). The dataset spans eight years (March 2008 to February 2016), meaning that two years of baseline data were collected prior to the first SHIP training in March 2010. In total, we observed 4374 practice-months of data across 46 practices and 96 months. Less than 1% of the data (42 practice-months) were missing.

### Intervention

The SHIP programme offers interactive training for general practice designed and led by GPs and practice nurses. During two afternoons of training for GPs (and three for nurses) SHIP focuses on providing essential knowledge and skills for HIV and sexual health care. The training updates factual knowledge of sexually transmitted infections and HIV.

SHIP is also designed to help attendees develop and apply relevant communication strategies and rapid sexual health risk assessment skills appropriate to different primary care clinical roles. Additional SHIP training in contraception for nurses is not reported on here.

SHIP had been conceived and initially developed in Birmingham, England, and was originally commissioned from the Heart of Birmingham National Health Service (NHS) Trust; SHIP had not been implemented in London before. SHIP sessions are taught by GP and practice nurse peer-educators, using a variety of methods. SHIP training in Haringey was commissioned intermittently during the 8 year observation period with two "fallow" periods, without training, of 12 and 23 months (Table 1).

**Table 1:** GP and practice nurse attendance at SHIP training March 2010 to end 2015.

**SHIP Commissioning**	**Testing data collection**	**Training period 1**						**FALLOW PERIOD 1 July 2012 to April 2013**	**Training period 2**			**FALLOW PERIOD 2 March 2014 to Nov 2015**	**Training period 3**
	2008–2010	2010			2011		2012		2013				2015
**Training round**	No training	Mar	May-June	Jan-Feb	Jun	Sept-Oct	Mar		June	Sep-Oct	Oct-Nov		Nov-Dec
**Total attendees**	N/A	44	42	29	34	19	14		22	18	22		18
	**Cumulative**:												
**GP completers**	N/A	11	14	11	8	5	0		6	6	11		88
**Cumulative GP completers**	N/A	11	25	36	44	49	49		55	61	72		80
**Practice nurse completers**	N/A	6	5	12	4	0	2		3	3	2		5

SHIP content outline is given in more detail in the S2 File. SHIP training centres on skills, notably, communication skills, needed to overcome the barriers to care, including those to HIV testing (Fig 1). These barriers have been elicited, collated and updated over more than a decade of training with contributions from GPs, practice nurses and SHIP peer educators.

### Measures

Our exposure was attendance at SHIP training. This was recorded on compulsory sign-in sheets at each training event and collected the participant’s name, role (GP or nurse) and the name of their practice. Our outcome measure was numbers of HIV tests by practice (number of tests from local laboratories is not available by individual doctor or nurse). HIV testing was chosen because it reflects a complex clinical behaviour, and is simple to measure, clinically meaningful, and subject to significant barriers to change. Laboratory HIV test numbers are an accurate measure of testing as rapid tests are not used by Haringey practices. It is near-impossible to artificially inflate practice HIV testing rates. HIV testing data were obtained directly from three laboratories responsible for processing all GP HIV test requests in Haringey (Whittington, North Middlesex and Homerton Hospitals), via Haringey Public Health. Laboratory staff removed duplicate positive results (same patient identifiers). We controlled for time and practice factors such as location and catchment area. The fixed-effect design of our analysis means that we did not need to collect observational data of our controls. This is further explained in the statistical analysis section.

### Statistical analysis

Our main outcome was the monthly count of HIV tests in each practice. We regressed this outcome on SHIP training exposure in Poisson regression analyses, controlling for practice fixed effects. The practice fixed effects controls for all time-invariant confounding factors at the level of the practice—i.e., factors such as practice location, practice catchment area and practice specialization.[25, 26] The month fixed effects control for time-varying factors affecting HIV-testing that are shared by all practices—i.e., factors such as HIV testing campaigns or changes in HIV testing guidelines, such as BHIVA and NICE guidelines in 2008 and 2011 respectively.[8–10] The control of time-invariant practice-level confounding, including those confounders that have not been observed, is the main reason that fixed-effects analyses are categorically different from many other observational study designs, which can only control for confounders that have been observed.[27, 28] The month fixed effects provide additional control for confounding time-varying factors. SHIP was commissioned in Haringey between March 2010 and December 2015, with two ‘fallow’ periods (Table 1). SHIP training is comprised of a total of two afternoons for GPs, and three for the nurses. 78 Haringey GPs and 42 practice nurses attended the training (estimated 40% and 30% of the total Haringey number, respectively).

## Results

### Attendance at training

**Fig 2:**
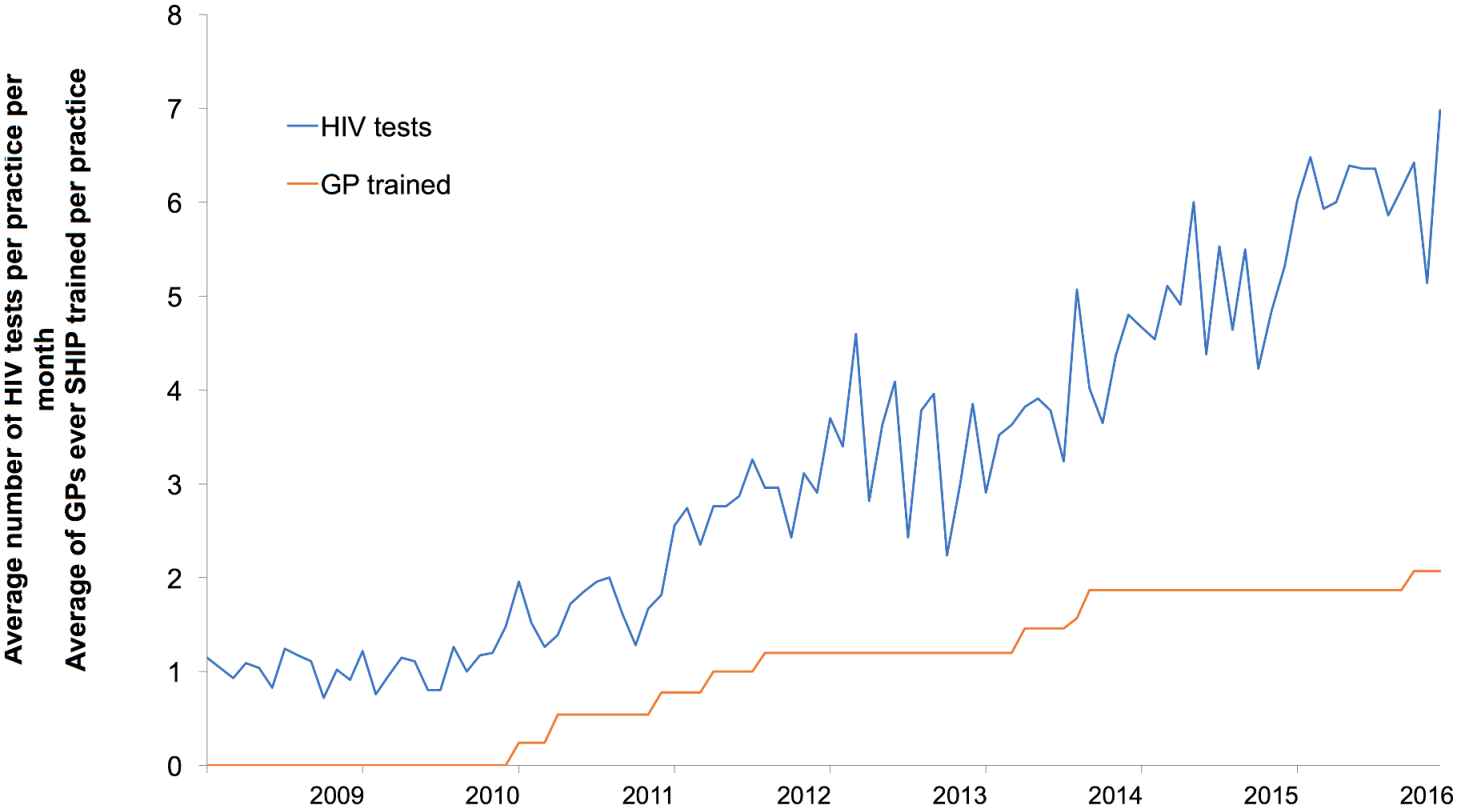
Average number of HIV tests and GPs trained per practice.

Fig 2 shows the average number of SHIP-trained GPs per practice per calendar month over the 8-year observation period, including two fallow periods. The first two years preceded the start of the SHIP intervention. By the final year an average of around two GPs per practice were trained. The average monthly HIV tests per practice increased six-fold, from approximately one to six over the eight-year observation period.

On average, for each additional GP completing SHIP training monthly practice HIV testing rates increased by 16% (Table 2). This effect was highly significant (p = 0.004). When we add the number of nurses who were SHIP trained to the regressions, the GP effect did not change substantially (testing rate ratio (TTR) 1.17, 95% confidence interval (CI) 1.06–1.29, p value 0.001); the nurse coefficient was not significant. When analysis was stratified by 2011 HIV prevalence in practice catchment areas, we found the increase in testing was driven by practices located in relatively high HIV prevalence areas ie >2 diagnosed per 1000 population (TRR 1.16, 95% CI 1.06–1.27, p value 0.002). Practices in low prevalence areas did not contribute to the effect (TRR 0.96, 95% CI 0.79–1.16, p value 0.658). To test whether the GP (or nurse) SHIP effects were decreasing or increasing with number of practice clinicians trained, we added higher-order polynomials of the GP and nurse variables to the regressions. None of these was significant; the relationship between SHIP-trained GPs and HIV testing rates was approximately linear: on average, each additional GP trained increases HIV testing rates by 16%.

**Table 2:** Effect of SHIP training on HIV testing rates. The unit of observation is practice-month. All regressions control for practice and month fixed effects. 95% CIs are based on robust standard errors, which were adjusted for clustering on practice. IRR = HIV testing incidence rate ratio, CI = confidence interval.

Additional GP trained	IRR	95% CI	p value	N Obser-vations	N groups	Wald χ^2^	Prob. χ^2^
All practices	1.16	1.05–1.28	0.004	4,374	46	44506	<0.001
Practices in catchment areas with HIV prevalence <2/1000	0.96	0.79–1.16	0.658	576	6	1599	<0.001
Practices in catchment areas with HIV prevalence ≥2/1000	1.16	1.06-1-27	0.002	3,798	40	5546	<0.001

### Testing for short-lived effects

The overall estimation of SHIP effects on HIV testing may be distorted by the greatest increases being in the earliest period after training. This is plausible, for example if motivation to adhere to training is highest shortly afterwards and then wanes over time. It is also possible that effects of training take time to establish—for instance, because training reduces the number of days spent in the practice and because taught content needs to be translated into practice processes. To test for such potential short term effects, we added an indicator variable to our main regression capturing whether a GP in a practice had been trained in the past 3 months (versus longer than 3 months ago). In a further distinct regression, we added an indicator variable to our main regression capturing whether a GP in a practice had been trained in the past 6 months (versus longer than 6 months ago). Table 3 shows the results of these two analyses. In both cases, the indicator variable for recent training was not significant and the coefficient size was close to one. Thus, the main SHIP effect estimate (the effect of an “additional GP trained”), remains nearly identical in both size and significant to the results without the variable controlling for short lived effects. Thus there is no evidence of any short term changes that differ from, and so distort, the identified long term effect of training.

**Table 3:** Testing SHIP training for short term effects.

	IRR	95% CI	p value	N Obser-vations	N groups	Wald χ^2^	Prob. χ^2^
Additional GP trained	1.16	1.04–1.28	0.006				
GP trained in the last 3 months	0.99	0.76–1.29	0.934	4,374	46	40771	<0.001
Additional GP trained	1.16	1.05–1.29	0.004				
GP trained in the last 6 months	0.95	0.75–1.20	0.658	4,374	46	33607	<0.001

The unit of observation is practice-month. All regressions control for practice and month fixed effects. 95% CIs are based on robust standard errors, which were adjusted for clustering on practice. IRR = HIV testing incidence rate ratio, CI = confidence interval

### Detection of HIV-positive cases

Overall an average of 1.42% of HIV tests were positive (95% CI 0.99–1.96%). In sub-analyses, we explored whether SHIP training increased this probability of diagnosis per test, as well as the positive detection rate per practice. For this analysis we used a linear probability model. This proportion did not increase significantly due to SHIP training and the GP SHIP training effect was nearly zero (0.001, 95% CI -0.003–0.005, p value 0.515). In line with this finding, GP SHIP training therefore increased the number of positive cases of HIV detected per practice per month to about the same extent (by 17%) as it increased the number of HIV tests per practice per month (positive detection rate ratio 1.17, 95% CI 0.93–1.48, p value 0.168). This result makes sense, because the positive detection rate is the product of the HIV testing rate and the positive detection ratio. However, the result was not significant, which was most likely due to a lack of power because positive HIV tests are, overall, rare events.

## Discussion

We have demonstrated here that a complex educational intervention, the SHIP training of GPs and nurses in London, had a highly significant, and sustained effect on practice HIV testing rates. SHIP training of each additional GP increased HIV testing rates by 16%. These results are plausible because in the SHIP training GPs identify opportunities to increase testing, in particular during individual consultations and in response to both clinical presentations and risk assessment. Our findings suggest that SHIP training is effective, both in terms of testing and in terms of absolute number of positive individuals identified.

In contrast to the GP effect, SHIP training of nurses did not affect HIV testing rates. Likely reasons for this finding include that GPs see more patients with symptomatic presentations, some of which may be HIV-associated. Furthermore, whilst training aims to increase HIV testing for asymptomatic patients found to be at risk, the workload of the nurses over-represents women. Men, including those who have sex with men (one of the main HIV-affected groups), are more likely to present to GPs rather than practice nurses. [29] In SHIP training for nurses, there is a strong focus on chlamydia testing, management of vaginal discharge, sexual health promotion and contraception. HIV testing rates are therefore unlikely to be the best outcome measure to assess the effect of SHIP training on practice nurses. In addition, only a comparatively small group of nurses were trained which could partially explain this null finding. The study team is currently collating data on testing and positives for much more common conditions (chlamydia and viral hepatitis) for the same 8 years of study, which will help evaluate whether nurse practice was affected.

One important finding of our SHIP evaluation is that increases in HIV testing were sustained. We found that the overall long term effect was not due to short lived changes in testing, such as those often observed in training interventions. [17, 20–22] In many training contexts, the motivation to adhere to training instructions is highest shortly after training. Over time, the training effects then wane because the people who were trained lose the motivation to adhere to new practice and forget the training contained. In contrast, our results indicated that the effect achieved in the periods (3, and also 6, months after training) are sustained in the long term. This finding suggests that changed clinical behaviours became normalised. The opportunity to measure effects over many years is rare for educational interventions, however even for shorter periods of follow up,[20–22] and broadening beyond sexual health, we were unable to find good evidence of the effect of educational interventions on clinical practice.

In stratified analysis, we found that the SHIP effects were largely explained by the effects in high HIV prevalence areas. High prevalence areas were defined as ≥2 cases /1000 in 15–59 year olds in the UK throughout the study period.[1] That the effect on testing rates was provided by practices in these areas is plausible because the need to test in high prevalence areas is emphasised by SHIP. We hypothesise that the range of strategies used by SHIP to help practitioners to identify and respond to relevant symptoms and also identify and respond to individuals at highest risk was of most relevance to practitioners in the high prevalence areas of the borough. Furthermore, we hypothesise that if a GP made a new HIV diagnosis (more likely in a high prevalence area), this would reinforce the changed clinical behaviours.

2015 Haringey GP HIV testing rates (by GP-registered population) compare favourably with those given in a 2016 Public England report.[30] This report, drawing on data from around half of all English practices in high prevalence areas, gives an average 64.9 tests per 10,000 registered population. In Haringey high prevalence areas this figure, for 2015, is 121.3 tests per 10,000 registered population (having increased from 21.5 in the first year of observation in 2008).

Another important finding of our study is that SHIP increased the positive detection rate to about the same extent as it increased the HIV testing rate. The positivity rate was high in Haringey, an average of 1.4%. By contrast, Health Protection England’s 2016 report on HIV testing[1] found average positive detection ratios in high prevalence areas to be 0.45% for GP HIV tests. We had expected that the positive detection ratio would decrease with increasing HIV testing rates because of a dilution effect: as GPs increased the number of people they test each month, positivity rates would decline. The sustained high positive detection ratio that is instead found—ie, the protection of the positive detection ratio despite increased HIV testing rates—may be due to a number of characteristics of the SHIP training which help GPs identify both asymptomatic and symptomatic patients with HIV.

While the SHIP effect on the detection of positive cases is important, testing that leads to the detection of HIV-negative individuals is also beneficial, in particular if it follows the determination of high HIV risk in a risk assessment (as opposed to population screening). Beyond reassurance for the patient, it may bring opportunities for patient education, Hepatitis B immunisation and referral for HIV pre-exposure prophylaxis (where available). In addition, testing behaviour may become more normalised (for individuals and within social networks).

We hypothesise that the listed features of SHIP that distinguish it from comparable interventions (Results) are likely to account for the effects. SHIP training is grounded in educational theory including tailoring to individual role;[31] and addressing barriers to change,[32] particularly those SHIP has identified that act in the consultation (Fig 1). When possible, performance feedback[33] is given to attendees of their individual practice HIV testing rates.

SHIP has a strong focus on communication skills including verbal strategies to help overcome the barriers to testing (Fig 1) and the use of rapid risk assessment to identify if testing is needed and help manage result-giving. These time-efficient communication skills feature positively in new peer-educator feedback who report adopting them as a change of practice. For further illustration of these please see the S4 File.

Our study design has a number of important strengths. First, the data we used were high quality, laboratory data. Second, our data were generated through routine data collection mechanisms and so the intervention and evaluation were carried out in a real-life setting. Thus, the artificiality of study context introduced by prospective controlled intervention studies was avoided. Third, because this was an audit of an educational intervention, the participants did not know that they were in a study, thus avoiding artificial testing results (although artificially inflated HIV testing rates are difficult to generate). The external validity of our findings is likely high, and higher than for a randomised controlled trial (RCT).

Fourth, the 8-year observation period allowed us to assess the long-term effectiveness of SHIP. Overall, the month fixed effects in our analysis control for all of time-varying confounding, i.e., background time trends that are shared by all practices. The study also controlled for in-practice spillover effect (e.g. if a colleague shared knowledge within their practice, the effect of this would be captured). The approach is also efficient: the costs of this study are essentially those of commissioning SHIP—as opposed to the much higher costs of implementing an RCT.

Our study also suffers from some important limitations. Firstly, training was offered on a ‘first-come-first-served’ basis, potentially limiting the generalisability to doctors that have (not yet) been exposed to SHIP training. However, the vast majority of practices in Haringey participated in this study, so that any selection effect threatening generalisability is likely small. Furthermore, only around 30–40% of Haringey GPs took part in the training, though this, if anything, is likely to result in an underestimation of the effect of the intervention. Secondly, the data did not account for any HIV tests in patients who were already aware of their HIV positive status, which could pose a threat to generalisability to geographical areas with lower or higher diagnosed prevalence. A further limitation is the inability to control for the spill-over effects which are not within the same practice, for example a trained doctor changing practice in the area. Any effect this had, however, would lead to an underestimate of the effect of SHIP.

HIV testing rates are a narrow measure of the effect of SHIP training. A more thorough evaluation would look for effect on testing for other STIs, including viral hepatitis and chlamydia.

### Comparison with equivalent interventions

We identified two educational interventions in sexual health in primary health care in the UK, the ‘Sexually Transmitted Infection Foundation’ (STIF) course and ‘3Cs and HIV’ both aiming to increase STI testing in the primary health care context. [21, 22] Similarly Joore describes a (substantially longer) educational intervention aiming to increase GP HIV testing rates in the Netherlands.[34] All of these interventions have published results showing they were relatively ineffective.[20–22, 34] We reviewed this published evidence, including process evaluation with hypotheses as to the lack of effect, where available. We also reviewed publicly available course materials and descriptions. Through this comparison we aimed to identify the features of SHIP that differ from the other interventions.

### Features of SHIP that differ from comparable interventions

Based on both published [20–22, 34] and publicly available course information and materials, we compared content of the SHIP intervention with others to identify differences that might explain the varying levels of effectiveness. Other interventions with similar objectives to the SHIP intervention and implemented in the UK also focused on relevant clinical content for education interventions and included participatory methods. Financial incentives to test, used in the ‘3Cs and HIV’ intervention (but not by SHIP), did not deliver change of practice. Exploring the lack of effect of ‘3Cs and HIV’ on chlamydia testing (data on HIV testing have not been published), several factors were identified.[20] These included poor adherence to intervention content; trainer support remaining unused; and computer prompts either not being applied or not appearing to have an effect. Finally, in 3Cs practices, chlamydia testing kits intended for use were in fact not readily available and the intervention videos and posters were not used. By comparison SHIP does not include many of these features including on-going trainer support, although it may create local champions through development of peer-educators; testing kits are not relevant to HIV testing based on venous sampling; and videos and posters are not offered either (although some patient resources are). The set up and use of computer prompts (promoted by ‘3Cs and HIV’) is mentioned only briefly in one nurse, but not GP, SHIP session: computer prompts would not be expected to overcome many of the barriers in Fig 1.

With respect to communication and verbal strategies the 3Cs intervention offered ‘model’ approaches to offer of a chlamydia test illustrated by video and recommended scripts (rather than experiential approaches to communication skill development and practice). [20, 21] Published information on 3Cs does not indicate it supports the use of routine brief sexual history taking and rapid assessment of risk. Joore explicitly considers the GPs expressed desire to use risk assessment to be a barrier, and to be discouraged. [17] This likely reflects Joore’s aim to increase HIV population screening (as opposed to increasing testing within individual consultations). By contrast the SHIP approach assumes that these skills are essential to help overcome barriers to HIV testing in individual consultations and to deliver higher quality care. This is illustrated in two brief clinical outlines the S4 File to illustrate how training may overcome some barriers, and see also Fig 1.

Through this comparison of content we identified distinguishing features, included in and central to SHIP training, that we hypothesise are important to change:

1 Taught, adaptable, communication skills for clinicians differentiating approaches for symptomatic, and asymptomatic patients (see S4 File):
  a Verbal strategies, such as introducing the topic of HIV
  b Rapid risk assessment for STIs and blood borne viruses
2 Precise tailoring to the general practice setting including relevant clinical presentations; clinical software support; primary care diagnostics and secondary service interface.
3 Separate teaching of GPs and practice nurses (as opposed to co-teaching)
4 Disguised repetition to enhance factual learning (eg individual HIV indicator conditions are each encountered in a number of different exercises)
5 Specific identification and listing of barriers to HIV testing by participants and trainers, with barriers crossed out if participants agree they have been addressed.

Additional factors not always commissioned / or deliverable in Haringey:

6 Performance feedback on individual practice testing rates
7 Invitation of HIV positive representatives to bring the ‘patient voice’
8 Support of training attendance with specific resources (such as locum payment).

SHIP can, and has been, replicated in different areas of the UK with funding and through collaboration and co-training (as occurred in Haringey). Further information on SHIP training is published elsewhere [23, 24]. However no current detailed ‘SHIP Implementation Handbook’ has been commissioned. It is not possible, without research funding, to state if such a handbook would be as efficacious as current approaches to implementation of SHIP which include centralised quality control (evidence updates and annual trainer days). SHIP is currently a not-for-profit Community Interest Company.

## Conclusion

SHIP is an educational intervention that produces a significant and sustained increase in HIV testing in primary health care for each GP trained in a high prevalence area of the UK. HIV tests used in routine clinical care in Haringey have a relatively high positivity. The finding that the HIV testing rate increased with no concomitant decline in the positivity suggests that SHIP training also contributed to the performance of GPs in detecting people with HIV. Unlike a randomised controlled trial, this evaluation is a ‘real life’ measure of the effect that commissioners of SHIP could expect should SHIP be implemented in a comparable area of the UK. SHIP effectiveness is likely explained by the components of SHIP training that distinguish it from interventions in the UK that did not demonstrate an effect of HIV testing.

Further analysis, applying the methods used here, of the effect on Hepatitis B and C testing rates and positives; diagnoses of chlamydia and gonorrhoea; and falls in use of the high vaginal swab, will give a better picture of the effects of SHIP on clinical practice, particularly in relation to nurses and to evaluate cost-effectiveness. Further, understanding of the mechanisms of action of SHIP would be aided by thorough process evaluation when SHIP is introduced into a new area. This may help identify the components of the training that have most effect on practice, and those that might be dropped.

## Supporting information

S1 File Key points in current UK clinical guidance on HIV testing that informs SHIP teaching content. (DOCX)

S2 File SHIP content outline. (DOCX)

S3 File SHIP evaluation in Haringey 2012 publication. (PDF)

S4 File SHIP clinical outlines. (DOCX)

S5 File SHIP de-intentified data. (XLSX)
