# Supplementary material for: Long term effect of primary health care training on HIV testing: A quasi-experimental evaluation of the Sexual Health in Practice (SHIP) intervention
Source: PLoS One. 2018 Aug 1;13(8):e0199891. doi: 10.1371/journal.pone.0199891 (PMC6070165; doi:10.1371/journal.pone.0199891)
Supplement: S1 File — (DOCX) [file pone.0199891.s001.docx]

**Appendix A**

**Key points in current UK clinical guidance on HIV testing that informs SHIP teaching content**

**[BHIVA and NICE guidance ^8-10^ ]**

Ensuring staff:

- emphasise that tests are confidential;
- are able to recommend HIV testing;
- are able to discuss HIV symptoms
- are able to recognise the symptoms of where primary HIV infection is a differential and recognise HIV indicator conditions (and able to offer and recommend an HIV test in these situations).
- are able to discuss the implications of a positive or negative test
- can provide appropriate information, advice and condoms to those who test negative
- are aware of local referral pathways – and can ensure patients are seen ‘preferably within 48 hours’

Ensuring services are staffed by people who

- are aware of, and sensitive to, the cultural issues facing black Africans
- are able to challenge the stigma of, and dispel myths surrounding, HIV and HIV testing

All health professionals should routinely offer and recommend an HIV test to:

- men and women known to be from a country of high HIV prevalence
- men and women who report sexual contact abroad or in the UK with someone from a country of high HIV prevalence
- patients diagnosed with an STI
- the sexual partners of men and women known to be HIV positive
- men who have disclosed that they have sexual contact with other men
- patients reporting a history of injecting drug use

‘Primary care providers should ensure annual HIV testing is part of the integrated healthcare offered to men who are known to have sex with men’

There is also guidance about repeat testing in a range of circumstances (including with respect to risks in the window period).
