# Supplementary material for: Long term effect of primary health care training on HIV testing: A quasi-experimental evaluation of the Sexual Health in Practice (SHIP) intervention
Source: PLoS One. 2018 Aug 1;13(8):e0199891. doi: 10.1371/journal.pone.0199891 (PMC6070165; doi:10.1371/journal.pone.0199891)
Supplement: S2 File — (DOCX) [file pone.0199891.s002.docx]

**Appendix B: SHIP Content Outline**

**SHIP Session Content Outline for General Practitioners’ Two afternoons**

**Afternoon 1: STI Update and Sexual History Taking***

Current evidence: scale of access of general practice by relevant groups.

Current evidence: many of those at high risk do not access specialist services.

Which genital infections and conditions are STIs? Which are not?

Characterise epidemiology, impacts on health, stigma, for each STI.

Testing and diagnosis in general practice; test performance; myth of the ‘full screen’.

Why distinguishing risk groups from risk behaviours matters in general practice.

Risk assessment questions [adapted overview partner history & condom use; pregnancy risk & attitudes; needles/injecting; holistic health approach to country of origin]

Learn and practice communication strategies for patients to:

- - - Destigmatise and normalise discussion of sexual health
    - Demonstrate non-judgmental and assumption-free approaches

Time-efficient verbal strategies allow clinicians to:

- - - Introduce the subject of sexual health in different clinical situations
    - Manage the patient at no apparent risk
    - Avoid ‘going round the houses’ (be time efficient)

Use of rapid risk assessment allows clinicians to:

- - - Be consistent with health promotion messages; educate and inform.
    - Identify those at high risk
    - Inform management of differential diagnosis in symptomatic patients
    - Avoid unneeded testing, advice, treatment, referral (be time efficient)
    - Anticipate those positives that will be truly unexpected to the patient

Clinical algorithms for diagnosis based on symptoms and risk, including safe syndromic management for vaginal discharge.

STI management, including when referral is indicated.

Partner notification myth-busting.

**Training for practice nurses takes 2 afternoons and differs by being tailored to their roles.*

**Afternoon 2: HIV** (from 2014 viral hepatitis added = Blood Borne Virus session)

Routes of transmission risk groups, risk behaviours for HIV.

Global and UK prevalence, the undiagnosed fraction.

Late diagnosis and impact on outcomes including mortality.

ARVs – a major success story.

Means of prevention, treatment as prevention, (latterly: PrEP).

Reflection on role of primary care re: testing, reducing late diagnosis, prevention.

Detailed local prevalence.

[*When feasible*: feedback testing rates in participants’ own practices]*.*

Identify barriers to HIV testing (and strike them out as learning proceeds).

Primary HIV infection: the ‘diagnostic jackpot’.

HIV-associated symptoms and presenting conditions.

Further practice of communication strategies (above) with sole focus on HIV

Local HIV services, meet the consultant, how to refer.

The patient’s story: diagnosis, living with HIV, role of health services (incl. primary care).
