# Supplementary material for: Long term effect of primary health care training on HIV testing: A quasi-experimental evaluation of the Sexual Health in Practice (SHIP) intervention
Source: PLoS One. 2018 Aug 1;13(8):e0199891. doi: 10.1371/journal.pone.0199891 (PMC6070165; doi:10.1371/journal.pone.0199891)
Supplement: S4 File — (DOCX) [file pone.0199891.s004.docx]

**Appendix C Two brief clinical Outlines**

**Clinical Outline 1: The patient with symptoms:**

**A 46 year old London born African Caribbean woman, Mrs P, known to her GP, consults with shingles. She is otherwise well.**

**Untrained GP: Dr M**

Dr M is aware that HIV is associated with shingles, but he thinks it unlikely from what he knows of his patient and her husband, and does not wish to cause alarm or appear inappropriate. The topic of HIV is not raised. Dr M reflects that HIV testing in practice is actually quite difficult.

**SHIP trained GP: Dr S**

Dr S is well aware that, should HIV prove to be the underlying cause, this might be the only opportunity to diagnose it before severe illness; there is an imperative to offer a test. Dr S knows that Mrs P comes from a higher prevalence population (‘risk group’) but has little information on risk behaviours; he knows that she is married. He uses verbal strategies to introduce the topic into of HIV into the consultation, causing some controlled, minimal concern. Dr S then introduces and performs a rapid risk assessment and identifies no apparent risk – Mrs P has described being in relationship that has been monogamous ‘from our wedding day’. Dr S then responds *“From what you have said, you are at little or no risk from HIV: would you like a test anyway?”*  Mrs P, confident in her marriage (and not undermined by Dr S), and reassured and informed by the process, agrees to test. Dr S feels more in control; if the test proves positive, at least he now knows it will be entirely unexpected to Mrs P and he can then manage result-giving accordingly. The test proves negative.

**Barriers overcome (Figure 1):** Dr S knows the benefits of HIV diagnosis and is becoming skilled and confident. He raised the topic using strategies that minimised alarm, stigma and embarrassment. He demonstrably avoided appearing racist or judgmental, or making assumptions, including of risk. His own concerns and fears for Mrs P of a positive result are more manageable when he has made a risk assessment. He feels he did a good job; it was time well spent. He knows he has preserved the doctor-patient relationship.

**Clinical Outline 2: The patient without symptoms**

**A 17-year-old man L**

**L attends for asthma review because he is planning gap year travel.**

**Untrained GP: Dr M**

Dr M is delighted to use the opportunity for a short consultation so she can catch up that morning. As L leaves, Dr M mentions chlamydia tests at reception, encourages L to ‘take care’ and offers him some condoms. Dr M also asks L to book a travel consultation with the nurse. L goes to pick up a chlamydia kit at reception but finds the waiting area full and changes his mind. He does however see the nurse the following week and has his travel immunisations. Dr M is pleased she remembered about the chlamydia test and condoms.

**SHIP trained GP: Dr S**

Dr S is aware that young people are more likely to need reassurances of confidentiality and also are at higher risk of sexually transmitted infections - as are international travellers. Dr S introduces the topic of sexual health into the consultation, and performs a rapid risk assessment. L is reassured by the non-judgmental and assumption-free approaches of Dr S.

Scenario 2a: L reports that he and his girlfriend are each other’s first sexual partners and are in a mutually monogamous relationship. The relationship became sexual just a few weeks ago, and they have used condoms very carefully. They intend to travel together. After risk assessment, Dr S states, “*From what you tell me, you are very good at looking after your sexual health. Would you like chlamydia or other tests anyway?*” L declines testing ‘for now’ but is interested in the opportunity to gain a little more information about condom use: the SHIP-trained nurse will also discuss contraception and emergency contraception when she sees him for his travel immunisations.

Scenario 2b: L has had sex with two young men of his age; on the last 3 occasions sex was unprotected. He is offered chlamydia, viral hepatitis and HIV tests (and information on window periods); condoms and lubricant; and rapid Hepatitis B immunisation (alongside other travel immunisations). When found HIV negative L is referred to a specialist sexual health service to discuss sexual health and PreP before he travels.

**Barriers overcome (Figure 1):** Dr S knows the benefits of STI diagnosis and preventive interventions. He demonstrably avoided assumptions of risk and gender of partner in the assessment. He avoided appearing judgmental or racist - or causing unnecessary alarm, stigma or embarrassment. Both versions of patient L are likely to be happy to see Dr S again if necessary in the future. Dr S is beginning to feel capable in sexual health, he feels his time was well spent (in either case).
